# Utilization of Secondary Eye Care Services in Western Kenya

**DOI:** 10.3390/ijerph16183371

**Published:** 2019-09-12

**Authors:** Hillary K Rono MMed, David Macleod, Andrew Bastawrous, Emmanuel Wanjala, Michael Gichangi, Matthew J. Burton

**Affiliations:** 1 International Centre for Eye Health, Clinical Research Department, London School of Hygiene & Tropical Medicine, London WC1E 7HT, UK; Andrew.Bastawrous@lshtm.ac.uk (A.B.); Matthew.Burton@lshtm.ac.uk (M.J.B.); 2 Kitale County and Referral Hospital, Box 98, Kitale 30200, Kenya; eswanjalah@yahoo.com; 3 MRC Tropical Epidemiology Group, London School of Hygiene & Tropical Medicine, London WC1E 7HT, UK; david.macleod@lshtm.ac.uk; 4 The Peek Vision Foundation, London EC2Y 9DT, UK; 5 Ophthalmic Services Unit, Ministry of Heath, Box 30016, Nairobi 00100, Kenya; gichangi58@yahoo.com; 6 Moorfields Eye Hospital NHS Trust, London EC1V 2PD, UK

**Keywords:** eye problems, eye health services, eye care utilization, routine hospital records, visual impairment and blindness

## Abstract

**Background:** Eye care provision is currently insufficient to meet the population’s eye health needs in Kenya. Many people remain unnecessarily visually impaired or at risk of becoming so due to treatable or preventable conditions. A lack of access and awareness of services are key barriers, in large part due to their being too few eye care providers in the health system for this unmet need. **Methods:** A hospital-based, retrospective analysis of patients who attended Kitale eye unit, Trans Nzoia County, Kenya from 1st January 2013 to 31st December 2015. Age and sex standardized hospital attendance rates by residence, age group, and sex were calculated for Trans Nzoia county and each subcounty. The changing trends in attendance rates were estimated by calculating the difference between base year and last year. Incidence rate ratios for attendance for each age-group, sex, and residence were estimated using a multivariable regression model. **Results:** 20,695 patients from the county were seen in Kitale Eye Unit in 2013, 2014 and 2015. In that period, 8.3% had either uncorrected refractive error, cataracts or glaucoma, the priority VISION2020 diseases, and 61.0% had allergic or other conjunctivitis or normal eyes, which could potentially be managed at primary eye care. During the study period, overall average annual attendance rate increased from 609 to 792 per 100, 000 population, incidence rate ratio (IRR) 1.30 (95% confidence interval (CI) 1.26–1.35). Attendance rates increased more in females than males (34.7% vs. 25.1%, respectively), IRR 1.07 (1.04–1.10). Attendance rates increased with increasing age, (highest among the elderly compared to the young). We found that in extreme age groups (>75 years and <15years) females were less likely to attend than males and there was reduced utilization from those based furthest from the hospital. **Conclusion:** Specialist eye services are heavily utilized by people with conditions that could be managed at the primary health care level. Barriers to accessing eye services were distance and gender, especially among the most vulnerable groups (young and the elderly). Integration of primary and secondary eye care services could lower barriers to essential eye care services to the population whilst lowering pressure on the limited specialist services by ensuring more appropriate utilization.

## 1. Background

About 36 million people are blind worldwide (visual acuity in the better eye < 3/60) and another 217 million are severely or moderately visually impaired (visual acuity in the better eye < 6/18) [1]. There is notable variation in the distribution of both blindness and Visual Impairment (VI) by region and gender. About 90% of people with blindness and VI live in low and middle-income countries (LMICs) such as Kenya [2]. Results from 11 population-based studies in sub-Saharan Africa suggest about 26 million people are visually impaired of whom, almost 6 million are blind [3]. The main causes of VI are uncorrected refractive errors (42%) and cataract (33%); both of these cause avoidable VI [3]. Recent studies have shown that the prevalence of VI is decreasing. However, women have a higher prevalence of blindness than men [4]. Despite the reduction in the prevalence of blindness, the number of people with VI has risen due to an increase in population and improved life expectancy [1].

There are multiple reasons for the high prevalence of VI in LMICs. Most of these relate to poverty and barriers to access and utilization of eye care services [5]. Poverty is a critical social determinant of VI, and in turn VI leads to further poverty [6]. For instance, as a result of low socioeconomic status, patients have no insurance, yet they may have to incur high transport costs to the hospital, which limits the use of services [7]. They are also likely to have low education, low awareness of the eye conditions, low awareness of the services available, and fear of adverse outcomes from treatment [8,9]. Additional barriers might include negative attitudes towards services and difficult communication between providers and patients [10]. Studies in LMICs, have shown that the need for eye services is high and people often travel long distances to access these services [11]. Communities with inadequate or inaccessible eye care facilities tend to seek other alternatives of eye care services, including self-medication, which may further contribute to VI and blindness [12].

Health system barriers include insufficient availability of services, shortages of health workers trained in eye care, inadequate skills of health workers and poor-quality eye care services [13]. An inverse relationship between need and provision of eye care services exists, especially in sub-Saharan Africa, meaning that there are fewer services available where the need is greatest, such as in rural areas [14]. The effect of these challenges in LMICs is suboptimal access and utilization of eye care services. Identifying barriers that hinder access and utilization of eye care services is therefore key in overcoming the burden of avoidable blindness [15]. 

The World Health Organization (WHO) recommends improving access and utilization of health services and monitoring equity as part of universal health coverage (UHC) [16]. Some of the interventions to improve access to eye care services in the literature include peer education, deployment of staff to rural areas, task shifting and integration of services, supervision of health staff, eliminating user fees and provision of health insurance [17]. However, the interventions should be selected based on identified gaps, which are likely to be context-specific. 

Few studies have however quantified the current utilization of eye services especially in Africa, therefore there is need to assess utilization of eye services so as to plan for efficient interventions and utilization. In this paper we report on the utilization of eye services in Trans Nzoia county, a county with an estimated population of 818,757 people in 2009 with one government-run secondary eye care unit, and three privately owned eye clinics. This evidence is useful for effective planning of services to reduce the burden of avoidable blindness.

## 2. Methods

A hospital-based, retrospective analysis of patients who attended Kitale eye unit, Trans Nzoia County, Kenya from 1st January 2013 to 31st December 2015 was conducted between June and October 2016. The study period was representative of standard practice as there were minimal non-surgical outreach services to the community from the hospital or other partners in this 36-month period; the eye unit at Kitale was operating optimally and it coincided with the final three years prior to devolution of Health services to the County governments in which considerable disruption to services was experienced.

**Figure 1:**
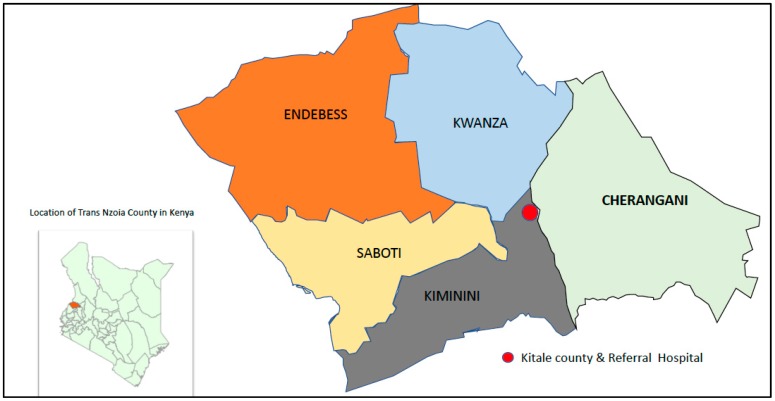
Location of Trans Nzoia County in Kenya and the sub-counties in relation and Kitale County & Referral hospital.

Trans Nzoia County is located 400 km West of Nairobi, Kenya. According to the 2009 census, Trans Nzoia County had a population of 818,757 people of which 49.7% were male, with an annual growth rate of 3.0% [18]. About 50.1% of the population live on less than one USA dollar per day, and 18.2% work for pay (employed) [19]. The county is subdivided in to five sub-counties, Figure 1: Kiminini (190,912), Cherangani (195,173), Saboti (174,956), Kwanza (166,524) and Endebess (91,192) [18]. To provide some indication of how far patients have to travel from each subcounty, the mean distance to Kitale hospital from health facilities in each county was calculated. Distance between the primary health facility and Kitale was estimated from Google-maps [20]. The mean distance and standard deviation (SD) of health facilities in the sub-counties to Kitale from the nearest to furthest are: Kwanza 14.9 kms (8.0), Kiminini 16.1 kms (7.4), Saboti 16.2 kms (9.6) Cherangani 18.2 kms (7.4) and Endebess, 29.9 kms (3.9).

Modified from a report by Kenya National Bureau of statistics et al. [19]. 

There are 146 health facilities of which 61 (41.8%) are government public facilities (6 hospitals, 12-health centers, 43 dispensaries), 22 (15.1%) are Faith-based/not for profit and 63 (43.2%) are private [21]. The doctor to population ratio is 5.4 per 100,000; Allied medical worker (clinical officers) is 9.6 per 100,000 and the nurse population ratio is 47 per 100,000 people. This is lower than the recommended WHO ratio of 230 per 100,000 population for any cadre [22]. Kitale Eye Unit is the only public eye unit in the county that provides secondary eye services daily at the central unit, training, and periodic services through mobile outreach at peripheral health centres [23]. The unit has one ophthalmologist, four ophthalmic clinical officers and eight nurses (two working outside the eye unit). Each consultation visit costs about one USA dollar while eye drops or surgery are subsidised but paid separately. Spectacles are not provided at the hospital.

Ethical approval was obtained from the London School of Hygiene & Tropical Medicine Medical Ethics Committee (Ref 10509) and Institutional Research and Ethics Committee (IREC) in Eldoret, Kenya (IREC/2016/40). Permission was sought from the hospital to use the secondary data. The study adhered to the Declaration of the Helsinki.

We obtained data from Kitale’s hospital database and the 2009 census report. All new patients who attended the Kitale eye hospital from 1st January 2013 to 31st December 2015 were included. We accessed the hospital attendance and morbidity Database (Med-boss) and extracted data for patients who attended Kitale County Hospital for consultations related to eye conditions for the 36-month period. Information extracted included age, gender, residence, date of first attendance, number of subsequent visits to the eye unit, diagnosis and visual acuity. Identifying information such as patient’s names and hospital numbers were deleted and a unique study number was assigned. Diagnosis was recorded based on the disease codes for routine reporting of eye diseases to the Ministry of Health, Kenya but reclassified using ICD10 classification [24]. The diagnosis recorded at first visit was used. Incomplete data such as those without location of residence, age or sex were excluded during analysis. 

Records of the health facilities and their catchment locations was obtained from the department of health in the county. The 2009 populations and census report were used to obtain the populations for Trans Nzoia and the sub-counties by age group and sex. An annual growth rate of 3% was assumed and that was applied uniformly across age groups, in order to estimate the population for the period 2013–2015.

Best presenting visual acuity (vision in the better eye) was used to assess visual status. Blindness was defined as Visual acuity (VA) < 3/60 in the better eye with available spectacle correction. Severe visual impairment was VA ≥ 3/60 to < 6/60, moderate visual impairment was VA ≥ 6/60 to < 6/24, mild visual impairment was VA ≥ 6/24 to < 6/12 and normal VA was ≥ 6/12. We compared visual acuity in males and females using an ordinal logistic regression model, adjusted for age, location and year.

We estimated the rate of attendance to the eye unit at the hospital per 100,000 population for the years 2013–2015. This was done overall for Trans Nzoia and also stratified by subcounty, age group, and sex. The rate was estimated by dividing the number attending the hospital during the year by the estimated population. The rates of attendance for individual diagnoses were also estimated.

The trends in standardized attendance rates for the corresponding time periods were expressed as the annual average percent change (AAPC). 

Analysis using the Poisson regression model was conducted using the standardized attendance rates as the dependent variable and the year of attendance to hospital as the independent variable. A Poisson regression model was conducted to evaluate the determinants of attendance adjusted for age, gender, residence and year of occurrence.

## 3. Results

### 3.1. Demographic Characteristics

A total of 24,776 patient records were extracted from hospital records on patients’ attending Kitale hospital for eye-related problems in the period 1st January 2013 to 31st December 2015. Of these 4081 (16.5%) patients were from outside Trans Nzoia county and therefore excluded, leaving 20,695 patients for analysis. 

Attendance to the Kitale general hospital in 2013 was 80,797, higher than the 57,127 in 2014 and 48,158 in 2015. In the eye department, attendance in 2013 was 5613 patients, fewer than in the subsequent two years (2014: 7336; 2015: 7746). Overall slightly more were women (52.4%) than Males, and about a third of patients were less than 15 years old. The mean age was 27.7 (SD 22.2), range (0–111) years. The subcounty where most patients came from was Kiminini (34.6% of all patients) whereas only 3.7% of patients originated from Endebess, the most remote of the five sub-counties, Table 1.

### 3.2. Visual Status of All Participants Attending Kitale Eye 

**Figure 2:**
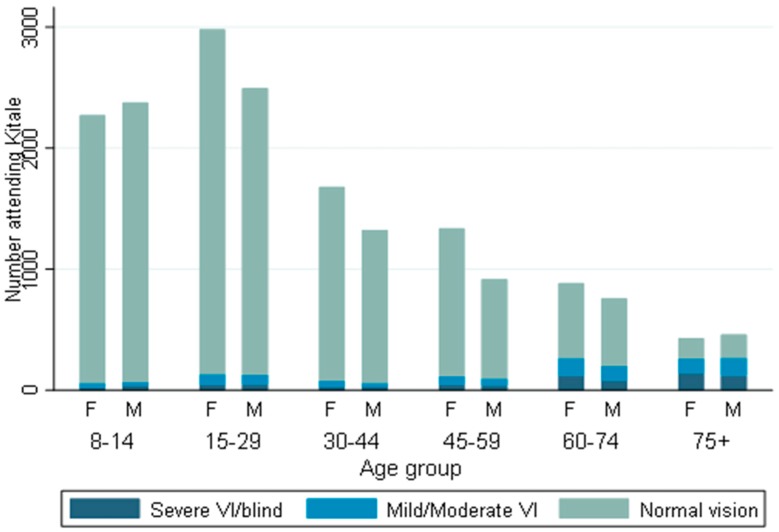
Visual status of patients attending Kitale Eye Unit between 2013 to 2015 stratified by age group and gender (M = male, F = female), (*N* = 17912).

Visual status of 17,912 patients were available. Information on vision from 2783 patients mainly children less than eight years old were not available because it was not measured. The majority of the patients had normal vision and were less than 30 years old, Figure 2. Visual impairment and blindness was common among those older than 45 years and increased with age. There was no evidence of a (OR 0.96, 95% CI 0.86–1.08, *p* = 0.521) difference in visual acuity between males and females.

### 3.3. Attendance Rates

**Figure 3:**
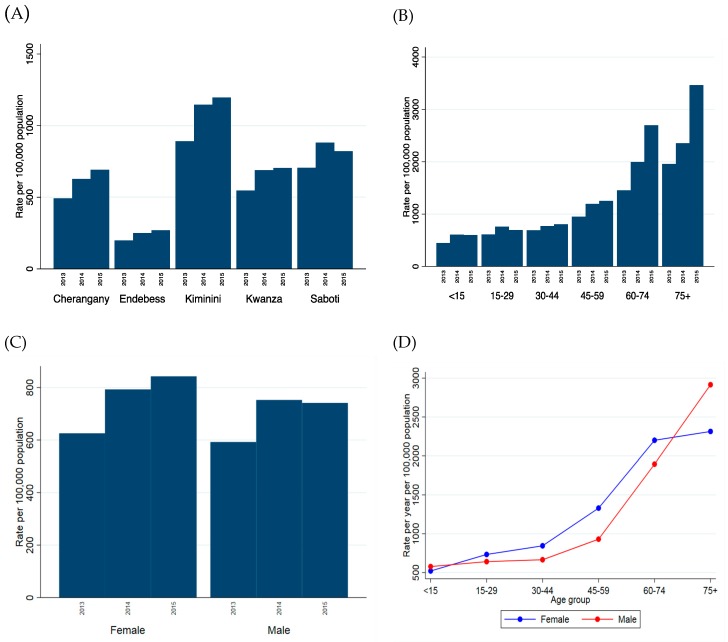
Attendance rates by (**A**) subcounty, (**B**) age group and (**C**) sex, across the three years. (**D**) shows the rate of attendance across age groups, stratified by age (attendance rate is the mean across the three years).

Overall, the annual attendance rate to Kitale eye unit increased by 30% over time, from 609 per 100,000 of the population attending in 2013 up to 792 per 100,000 in 2015. There was strong evidence (*p* < 0.001) that the rate of attendance differed by subcounty, with Endebess (the furthest from hospital) having the lowest attendance rate over the 3-year period and Kiminini (the nearest to the hospital) the highest, Figure 3A. In fact, the rate of attendance among individuals from Kiminini was estimated to be 4.5 times that of individuals from Endebess (controlling for age, sex and year), Table 2. There was also evidence (*p* < 0.001) that older aged groups had higher utilization of the eye unit services, Table 2, an increased attendance rate, and also the older age groups also showed the greatest increase in attendance across the three years, Figure 3B and Table 2. Overall, there was evidence that women attended at a higher rate than men, but the estimated increase was quite small (IRR 1.07, 95% CI 1.04–1.10, *p* < 0.001). There appeared to be little difference in 2013 but this gap widened in subsequent years, Figure 3C and Table 2.

It was noticed that the differences observed between men and women varied with age, and a test for interaction resulted in strong evidence of this (*p* < 0.0001). Although overall women attended at a higher rate than men, this difference was most prominent from the ages 30–74. Among those aged under 15 and those 75 and over, men had a higher estimated rate of attendance than women, Figure 3D and Table 3.

### 3.4. Attendance Rate and the Type of Eye Problem 

Over the three-year period just over 61% of people attending Kitale hospital for eye problems had conditions (allergic/other conjunctivitis or no eye problem found) considered suitable for management at the primary health care level, Table 4. Of the remaining conditions, the most common were eye injury (6.1%), post-surgical follow-ups (4.5%), refractive error/presbyopia (4.0%) and cataract (3.6%). The problems that showed the greatest increase in attendance rate were uveitis, glaucoma and post-surgical follow up for cataracts and glaucoma, all of which more than doubled between 2013 and 2015. There were decreases observed in the rate of attendance for people with no issues found and refractive error/presbyopia, especially between 2014 and 2015. The possible reasons are explained in the discussion.

Out of the 5793 school-age children (5–15 years), the proportion who had conditions suitable for management at the primary health care level is even higher, at 78.8% (allergic conjunctivitis 65.4%, normal 8.1% and other types of conjunctivitis 5.3%).

## 4. Discussions

The World Health Assembly has adopted a key resolution on Universal Health Coverage (UHC). Member states commit to provide access to necessary health services for the whole population, leaving no one behind [25]. Routine health information collected by eye health facilities or hospital reports within the national or regional health information system can provide a data source to monitor progress towards UHC [26]. To our knowledge, this is the first study to use routine hospital data to assess the utilization of eye services in Sub Sahara Africa. It has demonstrated the potential of such data in assessing equity, planning and monitoring eye health services delivery. 

This study was conducted in a county where most people live in rural communities and experience high inequality (poverty and unemployment) [19]. There was a decrease in attendance to the general hospital, probably due improvement of peripheral health facilities to handle most healthcare cases. In the same period, there was a gradual increase in the utilization of secondary eye care service from 609 per 100,000 population in 2013 to 792 by 2015, with higher utilization in children and women. When we compared the demographic characteristic of those who attended hospital to the population, we found higher attendance rates among residents who lived closer to the hospital, women and older people. Although, at both ends of the age range (vulnerable populations), male attend more than females. The increase did not overload the system because an additional ophthalmic nurse was posted to the unit to support increased workload. 

The higher absolute numbers attending among children was expected. This is in line with the structure of the Kenyan population (47.2% are <15 years) [19]. The attendance rates appear to be skewed in favor of male children, suggesting that male children could be more susceptible to eye ailments or preferential health-seeking behavior. The differences in health-seeking in gender has not been fully explained, but could arise from household power dynamics and prevailing social norms where men have authority in family decisions [12]. A review on barriers to utilization of eye care services found that women were more careful about their eye health than men, suggesting gender influence service utilization [27]. Gender disparities in accessing eye health care services were identified for eye trauma and cataract in studies from Tanzania where females had difficulty in accessing services [28,29]. In such communities, men decide on most matters affecting the family including those related to seeking health services [30]. This was observed in one study on access to cataract surgical services from Tanzania where women needed to seek permission from their husbands before going to hospital and out of fear of being a burden to the family, they opt live with the adversity [29]. 

We observed an inverse relationship in age distribution among those who attended hospital and the incidence of attendance by age. Cumulatively, there were more young people who attended hospital, Table 1, but when compared to stratum population, utilization increases with increasing age Figure 3B. We also found that the proportion of people with visual impairment (mild visual impairment and blind) increased with age and was more pronounced after the age of 45 years, Figure 2. The rise in the age stratum utilization can be explained by the various changes in the eye that occur with age and affect Visual function, such as complaints of glare in cases of nuclear sclerosis, presbyopia or reduced contrast sensitivity from progressive media opacities [31]. Other studies reported similar findings and attributed the increase to the higher prevalence of diabetes, hypertension, cataract, and related maculopathy that increase with age [32]. Higher absolute numbers of younger people attending is due to the pyramidal structure of this population [18].

We found that less than 1% of the people in Trans Nzoia had eye problems and presented for treatment at Kitale hospital. This was lower than findings of a community survey in Mbeere in Kenya, which found that 15.5% of the population reported at least one eye problem during six months prior to survey and had about 4.4% of the same population sought treatment from eye practitioners (health worker, doctor or optician) [33]. From our study we could not estimate the prevalence of the ocular morbidity in the population, however assuming that the population in Mbeere were similar then the current utilization does not meet current need. If Trans Nzoia county had a population of 1 million people, about 155,000 would be expected to have an eye problem of which less than 10,000 are currently accessing services from eye practitioners. The findings, therefore, suggest that a large proportion of people with eyecare needs are not reached; improving access to eye services is required. From this study, we could not establish where patients with eye problems sought treatment. However, a community survey on utilization of health services in other parts of Kenya, showed that patients who did not seek health services at the hospital largely resorted to self-medication by buying non-prescription drugs [12]. This finding might suggest that those with eye problems could be using alternative services. Another reason for low utilization is affordability of eye services [27], especially those from the low socioeconomic status. From this study, more than half of the population live on less than one USA dollar a day, which is equivalent to the cost of the consultation fee needed to access eye services at the hospital. When other hospital costs (eyedrops and surgery) are added, the cost of services become unaffordable for most people. Studies have reported that higher direct costs reduced the uptake of cataract surgery [34,35]. These costs increase further when indirect expenses such as transport and living costs of patients and those accompanying them to hospitals are included [36]. Most patients, therefore, may not be able to afford services, particularly those coming from rural areas [37]. 

Our study suggests greater distance to health facilities hinders access to eye health services among people in Trans Nzoia county. This finding is consistent with other studies from LMICs, which asked patients about barriers to attending health care services [8,12,38]. Distance not only affects access to health care by increasing indirect costs, but also determines the availability of transport [7]. In some other LMICs patients have been known to bypass local eye health services and go to tertiary services or wait for outreach eye services [39]. We do not think that this was the case here, because there were infrequent outreach services conducted in the main town and none in the rural areas. Besides, most parts of the Endebess subcounty is forested with Kitale being the nearest facility offering eye services. Since about 48.1% of residence in Endebess depend on subsistence agriculture compared to 30.5% in Trans Nzoia County and 32.7% in Kenya [19], it is possible that seasonal availability rather than lack of funds to be spent on health could be a factor because most residences grow maize, which takes up to nine months to mature. Also, few people (3.8% Endebess compared to 8.3% for Trans Nzoia County) have no work [19].

Diseases of the conjunctiva (allergic conjunctivitis and vernal keratoconjunctivitis) were the most common problem. This finding is similar to other studies in Kenya which have reported allergic conjunctivitis to be the most common problem [33,40]. We found that 61% of the people utilizing eye services had eye conditions (allergic conjunctivitis, other conjunctivitis, and no eye health problem) that could have been managed in primary health facilities (dispensaries and health centres), particularly among the school going age group. According to Kenya’s strategic plan for eye care, secondary care eye facilities are equipped to manage Vision 2020 priority diseases (cataracts, glaucoma, corneal diseases, and refractive errors) that are responsible for the majority of visual impairment [41]. We found that only 8.3% of the patients seen at the hospital had these priority conditions (cataracts, glaucoma or refractive errors) suggesting a mismatch in the utilization of available capacity. The decline in utilization of eye services by people with refractive errors could have been due to lack of spectacles at the eye unit resulting in the patients seeking the services from other eye care providers. Encouragingly, during these three years, there was increasing utilization of eye services at Kitale by people with potentially blinding conditions such as those with diabetic retinopathy and those on post-surgical follow-up for cataracts. There was also a reduction in people without any eye problem (normal). This may have been attributed to improved accuracy of referrals following refresher training of health workers at the health posts (dispensaries and health centres) on how to identify and refer eye people with problems that took place from September 2013 to June 2014, and funded by Operation Eyesight Universal (OEU) and Seeing is Believing. Monthly Continuous Medical Education sessions provided by eyecare workers may have contributed [42]. Studies from the region identified skills of the general health workers in Primary Eye Care (PEC) to be low (about 8.2% were able to measure visual acuity) [43], however short training, supervision and continuous medical education for the PEC workers could improve their skills leading to better utilization of available eye services [44]. Training and deployment of middle level eye care workers (ophthalmic nurses and ophthalmic clinical officers) to primary eye care facilities could also improve management of these eye conditions. In some countries, this carder provides the bulk of eye care (including preventive, diagnostic and referral services) especially in rural areas [45]. 

Overall, secondary eye care services in Kitale were utilized by people closer to the hospital and by many people with conditions that could have been managed at the primary care level. This can result in over loading the limited central eye care services and further increasing the barriers to those with priority eye conditions. This data suggests a need to rethink the structure and mode of delivery of eye care services. There is potential for greater task shifting and integration of simpler services into primary health care [17]. To overcome the barrier presented by distance to the health facility there is a role for deploying eye care staff to provide regular outreach services, integrated with the standing primary health services further developing both capacity at the primary level in tandem with providing ad hoc secondary services [39,46].

### Limitations of the Study

We had incomplete or missing records. We assumed linear population growth, as provided for in the census to extrapolate the population size for subsequent years but this estimate may not be accurate. The study did not capture information on the non-users of public health services or those who are treated elsewhere.

## 5. Conclusions

In conclusion, secondary eye services in Kitale Eye Unit, Trans Nzoia were heavily utilized by people with conditions that could be managed at in a primary care setting. Barriers to accessing services were distance and gender especially among the most vulnerable groups (young and the elderly). We recommend, that the eye health services be redesigned to increase access to community and primary level services, thereby reducing inappropriate utilization of secondary level services and reducing the barriers to access to increased secondary service capacity with a particular focus on equitable access for the young, old and those living at greater distances from secondary level care. Further population-based studies to assess ocular morbidity and the barriers to eye service in the community; as well as changing trends in utilization of eye services are recommended. Similarly, qualitative studies to explore the barriers to utilization of the services.

## Figures and Tables

**Table 1:** Characteristics of Patients attending Kitale eye unit between 2013 to 2015 (*N* = 20,695)**.**

Characteristic	*N* (20,695)	%
Year of attendance (*N* missing = 0)		
2013	5613	27.1%
2014	7336	35.5%
2015	7746	37.4%
Subcounty (*N* missing = 0)		
Cherangani	4111	19.9%
Endebess	761	3.7%
Kiminini	7178	34.6%
Kwanza	3755	18.1%
Saboti	4890	23.6%
Age group (*N* missing = 1)		
<15	7389	35.7%
15–29	5496	26.6%
30–44	3016	14.6%
45–59	2258	10.9%
60–74	1645	8.0%
75+	890	4.3%
Sex (*N* missing = 839)		
Female	10,403	52.4%
Male	9453	47.6%

**Table 2:** Attendance rates to Kitale eye unit for eye consultations, by year, subcounty, sex, and age in Trans Nzoia Kenya.

Characteristic		Attendance Per 100,000 of the Population			% Change 2013 to 2015	Incidence Rate Ratio IRR(CI) *	*p*-Value
		2013	2014	2015			
Year	2013	609.1	-	-	30.1%	Baseline	<0.001
	2014	-	772.9	-		1.27 (1.23–1.31)	
	2015	-	-	792.3		1.30 (1.26–1.35)	
Subcounty	Cherangani	492.6	627.2	690.8	40.3%	Baseline	<0.001
	Endebess	197.8	249.7	270.0	36.5%	0.40 (0.37–0.43)	
	Kiminini	890.8	1146.3	1196.3	34.3%	1.78 (1.72–1.85)	
	Kwanza	546.9	689.0	704.1	28.7%	1.07 (1.02–1.12)	
	Saboti	705.4	880.6	820.9	16.4%	1.33 (1.27–1.38)	
Sex **	Male	592.4	752.7	741.4	25.1%	Baseline	<0.001
	Female	625.6	792.8	842.7	34.7%	1.07 (1.04–1.10)	
Age group	<15	447.7	605.1	597.0	33.4%	Baseline	<0.001
	15–29	612.8	759.9	693.9	13.2%	1.25 (1.21–1.29)	
	30–44	688.8	772.6	805.7	17.0%	1.37 (1.32–1.43)	
	45–59	951.3	1193.8	1252.8	31.7%	2.06 (1.96–2.16)	
	60–74	1451.9	1996.0	2697.0	85.8%	3.74 (3.54–3.94)	
	75+ years	1954.6	2354.5	3463.0	77.2%	4.71 (4.39–5.05)	

* All IRR estimates adjusted for year, subcounty, sex and age group; ** 839 individuals were missing sex. It was assumed that 50% of the missing were male and 50% female.

**Table 3:** Stratum specific Incidence Rate Ratios (IRR) for age and sex.

Strata	Exposure	Incidence Rate Ratio IRR (95% CI)	*p*-Value
	Sex (Male is baseline)		
<15	Female	0.90 (0.86–0.94)	<0.001
15–29	Female	1.14 (1.08–1.20)	<0.001
30–44	Female	1.26 (1.18–1.36)	<0.001
45–59	Female	1.42 (1.31–1.55)	<0.001
60–74	Female	1.16 (1.05–1.28)	0.003
75+	Female	0.79 (0.69–0.90)	0.001
Female	<15	Baseline	<0.001
	15–29	1.41 (1.34–1.48)	
	30–44	1.62 (1.53–1.72)	
	45–59	2.55 (2.39–2.72)	
	60–74	4.24 (3.93–4.56)	
	75+	4.46 (4.03–4.92)	
Male	<15	Baseline	<0.001
	15–29	1.11 (1.05–1.17)	
	30–44	1.15 (1.08–1.22)	
	45–59	1.61 (1.50–1.73)	
	60–74	3.29 (3.04–3.55)	
	75+	5.05 (4.59–5.57)	

* *p*-value for interaction is <0.0001.

**Table 4:** Trends and percentage change in attendance rates to Kitale Eye Unit (standardized to population for respective year) by eye conditions in Trans Nzoia County.

Diagnosis	Total Attending 2013–2015 (%)	Attendance Rate Per 100,000 Population			
		2013	2014	2015	% Change 2013 to 2015
Normal	1895 (9.2%)	95.9	76.8	28.8	−69.9%
Cataract	747 (3.6%)	19.1	25.9	33.2	74.1%
Refractive errors & Presbyopia	820 (4.0%)	36.5	36.0	14.5	−60.2%
Glaucoma	150 (0.7%)	3.4	3.5	8.8	161.5%
Allergic conjunctivitis	9245 (44.7%)	268.1	338.1	364.7	36.0%
Conjunctivitis-other	1459 (7.1%)	42.4	57.1	53.8	26.8%
Corneal diseases	354 (1.7%)	10.4	10.7	16.0	53.2%
Retinal diseases	708 (3.4%)	18.9	26.0	29.4	55.5%
Eye injury and FB in eye	1271 (6.1%)	32.9	39.0	61.2	86.0%
Uveitis	444 (2.2%)	8.0	15.4	22.9	185.3%
Conjunctival growths	695 (3.4%)	17.3	24.2	31.3	81.4%
Chalazion and other lid swellings	358 (1.7%)	8.5	13.7	15.3	81.3%
Lid inflammations	67 (0.3%)	2.0	2.2	2.9	46.6%
Others	1548 (7.5%)	24.7	79.0	58.3	135.6%
Post-surgical follow-up	934 (4.5%)	21.1	25.2	51.2	143.4%
